# Hemocyte-Secreted Type IV Collagen Enhances BMP Signaling to Guide Renal Tubule Morphogenesis in *Drosophila*

**DOI:** 10.1016/j.devcel.2010.07.019

**Published:** 2010-08-17

**Authors:** Stephanie Bunt, Clare Hooley, Nan Hu, Catherine Scahill, Helen Weavers, Helen Skaer

**Affiliations:** 1 Department of Zoology, University of Cambridge, Downing Street, Cambridge CB2 3EJ, UK

**Keywords:** DEVBIO

## Abstract

Details of the mechanisms that determine the shape and positioning of organs in the body cavity remain largely obscure. We show that stereotypic positioning of outgrowing *Drosophila* renal tubules depends on signaling in a subset of tubule cells and results from enhanced sensitivity to guidance signals by targeted matrix deposition. VEGF/PDGF ligands from the tubules attract hemocytes, which secrete components of the basement membrane to ensheath them. Collagen IV sensitizes tubule cells to localized BMP guidance cues. Signaling results in pathway activation in a subset of tubule cells that lead outgrowth through the body cavity. Failure of hemocyte migration, loss of collagen IV, or abrogation of BMP signaling results in tubule misrouting and defective organ shape and positioning. Such regulated interplay between cell-cell and cell-matrix interactions is likely to have wide relevance in organogenesis and congenital disease.

## Highlights

► Tubule morphogenesis is guided by a dynamic subset of leader cells ► Blood-cell-derived collagen around the tubules is required for morphogenesis ► Collagen potentiates signaling through BMP for tubule morphogenesis ► Morphogenesis depends on BMP signaling in tubule leader cells

## Introduction

Organ shape results not only from regulated cell division and growth, but also from the changes in cell adhesion, shape, and movement that sculpt tissues, bringing the cells of specific types into precise three-dimensional relationships. In addition, organs lie in the body cavity in reproducible positions with respect to each other and to the vascular and nervous systems that service them and regulate their activity. Analysis of the morphogenesis of organs has revealed regulatory mechanisms of two kinds: those that are intrinsic to the tissue (such as planar cell polarity in notochord extension; Jiang et al., 2005), the orientation of cell divisions (Baena-Lopez et al., 2005), and changes in cell shape (Myat and Andrew, 2000), and extrinsic mechanisms, involving interactions with other tissues (such as guidepost cues; Englund et al., 2002; Kato et al., 2004; Kolesnikov and Beckendorf, 2005). The final arrangement of tissues depends on the exchange of signals and cellular responses to these guidance cues ensure the correct topology both of tissues within an organ and of organs within the body cavity.

Such tissue interactions have been studied extensively in the developing nervous system, where axon guidance cues dictate neuronal projections and the formation of specific connections (Chilton, 2006), and in the control of branching morphogenesis, where dynamic local gradients of signals such as FGF from enveloping mesenchymal cells pattern the initiation and elongation of epithelial buds (Ghabrial et al., 2003; Hogan and Yingling, 1998). These studies have revealed remarkable conservation in the signaling pathways involved and in the response of cells to them (Dickson, 2002; Metzger et al., 2008). In contrast relatively little is known about the cues that control the final three-dimensional shape and positioning of organs. The outgrowth of the salivary glands and positioning of tracheal arbors in *Drosophila* provide model systems which show that cells respond to local signals from overlying tissues; the developing salivary glands respond to cues from the visceral mesoderm (Bradley et al., 2003) and specific tracheal branches respond to BMP, EGF, and Wnt signals (Cela and Llimargas, 2006; Kato et al., 2004; Shaye et al., 2008). In an attempt to further our understanding of the tissue interactions that regulate organ positioning, we have analyzed the cues that guide the outgrowth and positioning of *Drosophila* renal tubules.

*Drosophila* renal (Malpighian) tubules are suspended in the hemolymph of the body cavity and maintain homeostasis by osmotic, ionic, and acid/base modification through active transport processes, and by the secretion of toxic waste (Dow, 2009; Maddrell, 1971). As insects have an open circulatory system, effective excretion depends on local sampling and removal of waste. Accordingly, the tubules course through the body cavity to maximize hemolymph sampling; in *Drosophila* two of the four tubules extend into the anterior body cavity and the other pair projects posteriorly. As they grow, the four tubules take up remarkably stereotypical positions.

In this paper, we analyze factors that regulate the pathfinding of the tubules as they extend. Focusing on the anterior pair of tubules, we show that PDGF/VEGF-mediated interactions between the tubules and migrating hemocytes are required for normal tubule pathfinding. We show that hemocytes secrete type IV collagen around the growing tubules and that this basement membrane component is critical for the sensitivity of a subset of tubule cells to local sources of the BMP ligand, Decapentaplegic (Dpp), which act as guidepost cues to promote forward projection of the tubules. In the absence of hemocytes or of collagen IV, BMP pathway activation fails in tubule cells and the anterior tubules do not project anteriorly. We suggest that collagen IV acts to enhance ligand presentation to tubule cell receptors and is required to ensure ligand-mediated activation of the BMP pathway.

## Results

**Figure 1:**
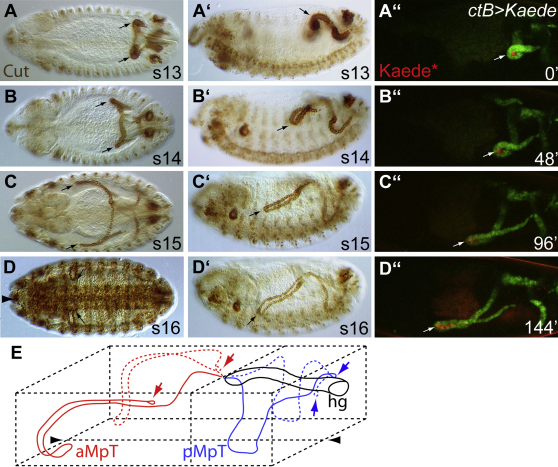
Malpighian Tubule Morphogenesis Is Highly Reproducible (A–D) Wild-type embryos stained for Cut (brown) to mark the tubules as they elongate, coursing through the body cavity to take up stereotypical positions by stage 16. (A and A′) Stage 13. The four postmitotic tubules start to elongate by cell rearrangement. The anterior tubules bend forming a “kink” region, which projects toward the head (A and A′, arrows). Stage 14 (B and B′) and 15 (C and C′). The anterior tubule kink projects anteriorly on either side of the midgut (B and B′, arrows), and drops ventrally (B′ and C′, arrows). (D and D′) Stage 16. The kink regions of all four tubules lie ventrally, extending toward the midline on either side of the CNS (D and D′, arrows, anterior; midline, large arrowhead in D). (A″–D″) WT embryos expressing the photoconvertible fluorophore Kaede in the tubules (green). Kaede was activated in the kink region at time 0 (^∗^Keade, red). Snapshots of a living embryo at times indicated reveal labeled cells in the kink region up to 96 minutes. (E) Diagram to show the invariant positions adopted by the tubules in the embryonic body cavity by stage 16. Arrowheads, ventral midline. hg, hindgut; aMpT, anterior MpT; pMpT, posterior MpT. Anterior is to the left and dorsal is to the top in lateral perspectives (A′–D′). See also Movie S1.

The four renal tubules bud out from the embryonic hindgut during stage 10 of embryogenesis and grow through cell division until stage 13 (Figures 1A and 1A′) (Denholm and Skaer, 2005). All further growth is postmitotic, occurring through increase in cell size accompanied by endoreplication (Edgar and Orr-Weaver, 2001), by cell rearrangement, to convert the short thick tubules into long thin ones (Figure 1), and finally through changes in cell shape, as the epithelial cells acquire their mature squamous morphology (Skaer et al., 1987). As the tubules elongate through convergent-extension cell rearrangements, they follow a highly reproducible course through the body cavity. The anterior tubules bend back on themselves so that the apex of this bend (the “kink region”, arrowed in Figure 1) of each tubule extends forward just below the leading edge of the closing epidermis (Figures 1B and 1B′), on either side of the developing midgut (Figures 1C and 1C′). The kink regions then dip ventrally while the distal tips extend to retain their dorsal position (Figures 1D, 1D′, and 1E).

We followed cells in the kink region of the anterior tubules in real time to assess whether cells in this region of the tubules represent a stable population during morphogenesis. Movies (see Movie S1 available online) indicate that some cells do remain in this region as the tubules elongate and navigate past the midgut. To confirm this impression, we photoconverted (green-to-red) cells in the kink region of tubules labeled with the fluorophore Kaede (Grueber et al., 2007) from early stage 13. We find that red cells remain in the kink until stage 15 (Figures 1A″–1C″), but that they are not a static population as they later move out of the kink into more proximal regions of the tubules (Figure 1D″).

**Figure 2:**
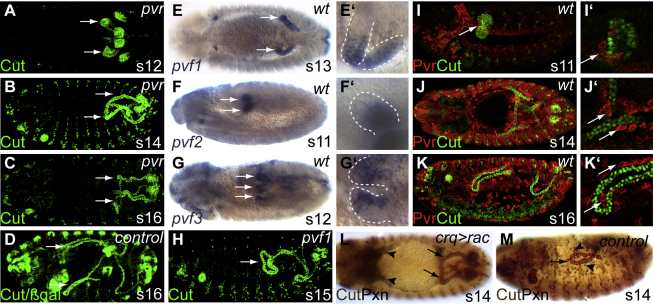
PDGF/VEGF Signaling Is Required for Anterior Tubule Morphogenesis (A–D) In *pvr* mutant embryos the anterior tubules fail to project forward. (A) Although specified normally (A, stage 12, tubules stained for Cut, green), anterior tubules misroute into the posterior of the embryo (B, stage 14; C, stage 16). (D) stage 16, sibling control. (Arrows; anterior tubules.) (E–G) in situ hybridization for PDGF/VEGF ligands, Pvf 1, 2, and 3. (E) *pvf1* is expressed in the kink and distal regions of anterior tubules from stage 13 (arrow). (F) *pvf2* is expressed from stage 11 (arrows in F) to 14, in the proximal region of all tubules. (G) *pvf3* is expressed in the distal region of all tubules from stage 11 to 14 (stage 12 tubules arrowed) (E′–G′) magnified views of tubules (dashed white lines) in (E)–(G). (H) In *pvf1*^G0246^ mutants anterior tubule forward projection is stalled (arrow, stage 15). (I–K) The PDGF/VEGF receptor Pvr (stained in red) is expressed in migrating hemocytes; some associate with developing tubules (Cut, green). (I) stage 11. A few hemocytes associate with tubule primordia (I′, arrow), continuing as the tubules extend (J, stage 15; K, stage 16; J′ and K′, arrows). (L and M) Expressing constitutively active Rac in the hemocytes (*CrqGal4, UAS-rac^V12^*) stalls hemocyte migration, preventing association with the developing tubules. Hemocytes (Peroxidasin, black) remain in the anterior (L, arrowheads) and the anterior tubules (Cut, brown) fail to extend toward the head (L, arrows). Compare with sibling control (M). Anterior is to the left and dorsal is to the top in lateral perspectives. See also Figure S1 and Movie S2.

We reasoned that tubule outgrowth might be regulated as they course through the body cavity by “guidepost” signals, which promote or inhibit extension into specific domains. Focusing on anterior tubules, we therefore screened mutants defective in signaling for tubule misrouting. In embryos lacking the receptor for the *Drosophila* platelet-derived growth factor/vascular endothelial growth factor (PDGF/VEGF) pathway (Duchek et al., 2001; Heino et al., 2001), the anterior tubules fail to grow into the anterior but instead extend backward with the posterior tubules (Figures 2A–2C; compare with Figures 1E and 2D). We used in situ hybridization against the three *Drosophila* PDGF/VEGF ligands (Pvf1-3) to assess which tissues express them and found that a major source of all three was the renal tubules themselves (Figures 2E–2G and S1A–S1G) (see Cho et al., 2002; Wood et al., 2006). *pvf1* is strongly expressed in just the anterior tubules from stage 12, persisting throughout tubule elongation (Figures 2E, 2E′, and S1A–S1C). *pvf2* is expressed in the proximal and *pvf3* in the distal regions of all four tubules from stage 11 to 14 (Figures 2F, 2F′, 2G, 2G′, and S1C–S1G).

As *pvf1* is expressed in the anterior but not posterior tubules, we assessed the tubule phenotype in *pvf1^G0146^* embryos. The anterior tubules fail to extend forward, instead coiling up close to their site of origin from the hindgut (Figure 2H). We confirmed the specificity of the tubules as a source of ligand by driving *pvf1*-RNAi with a tubule-specific Gal4 driver. This results in the same misrouting phenotype (Figures S1H and S1I).

The PDGF/VEGF receptor (Pvr) is prominently expressed in the developing hemocytes (Cho et al., 2002; Heino et al., 2001), initially in bilaterally paired clusters of mesodermal cells in the head (Figure 2I) and subsequently as they migrate out to populate the body cavity (Figures 2J and 2K). Pvr-expressing cells cross into the posterior region of the extended germ band and cluster around the primordial buds of the Malpighian tubules at stage 11 (Figure 2I′). A subset of Pvr-positive cells continues to be associated with the tubules as they grow and elongate (Figures 2J′ and 2K′). We demonstrated that these tubule-associated, Pvr-expressing cells are indeed hemocytes by double labeling for Pvr and Croquemort, a hemocyte marker (Franc et al., 1996) (Figures S1J and S1K). Live imaging reveals that the interaction between the hemocytes and the tubules is highly dynamic. There do not appear to be tubule-specific hemocytes; rather, associations are short lived and may be repeated (Movie S2).

Because Pvr is widely expressed, we confirmed the significance of the tubule:hemocyte association for anterior tubule morphogenesis by stalling hemocyte migration using Gal4-induced expression of a constitutively active form of the small GTPase, Rac, in the hemocytes. Expression of Rac^V12^ prevents the formation of filopodia and lamellipodia, which are essential for hemocyte migratory activity (Paladi and Tepass, 2004; Stramer et al., 2005). The hemocytes remain clustered in the head and the anterior tubules extend posteriorly (Figures 2L and 2M) as they do in *pvr* mutant embryos (Figures 2B and 2C). We confirmed that activation of the receptor is required specifically in the hemocytes for normal anterior tubule morphogenesis by driving a dominant-negative Pvr using the hemocyte-specific driver, *glial cells missing*Gal4 (Figures S1L and S1M). Thus Pvr-mediated guidance of hemocytes to the outgrowing tubules is required for their normal pathfinding through the body cavity.

We next asked what the role of the hemocytes might be in directing the forward extension of the anterior tubules. The dynamic nature of the association between the hemocytes and tubules (see Movie S2) argues against direct tractive activity whereby the tubules might be anchored or pulled along by the migrating hemocytes. However, hemocytes are active secretory cells that lay down components of the extracellular matrix (ECM), particularly the basement membrane (BM) (Fessler and Fessler, 1989; Wigglesworth, 1939; Wood and Jacinto, 2007). We therefore analyzed the expression of BM components focusing on the tubules and on the hemocytes associated with them. Using in situ hybridization, we followed the expression of four key molecular classes that are found in diverse basement membranes: collagen IV, laminin, perlecan-like proteoglycans, and nidogen/entactin (Hynes and Zhao, 2000).

**Figure 3:**
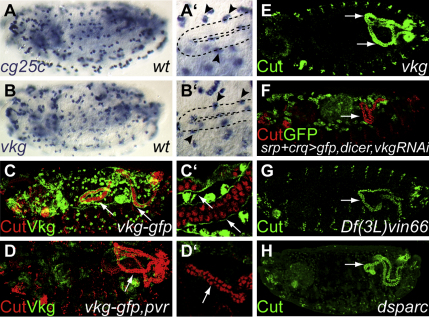
Hemocyte-Deposited Collagen IV Is Required for Tubule Morphogenesis (A and B) There are two collagen IV genes in *Drosophila*, *cg25c* (A) and *viking* (*vkg*, B). In situ hybridization shows that both are expressed in hemocytes (A′ and B′, arrowheads, stage 15 anterior tubule outlined). (C and D) Viking (green) is secreted by the hemocytes and forms an extracellular sheath around the elongating tubules (arrows in C and C′. Stage 15 embryo stained for Cut, red). In *pvr* embryos, the hemocytes do not migrate and the extracellular sheath of collagen around the tubules is not deposited (arrows in D and D′, stage 15 embryo). The anterior tubules project posteriorly (D). (E–H) Disrupting the synthesis or deposition of collagen IV perturbs tubule morphogenesis. The forward projection of anterior tubules is stalled in *vkg^K00236^* embryos (E, late stage 14, arrows). Knocking down collagen IV specifically in the GFP-expressing hemocytes by RNAi results in the same anterior tubule pathfinding defect (F, stage 15, arrows, see also Figures S1J and S1K). Forward projection also stalls in *Df(3L)vin66* embryos, in which the lysyl hydroxylase enzyme encoded by *CG6199* is deleted (G, stage 15, arrow), and in *dsparc* mutants, in which collagen IV secretion is lost (H, stage 15, arrow). Anterior is to the left and dorsal is to the top in lateral perspectives.

In *Drosophila* two genes encode collagen IV α chains, *viking* (*vkg*) and *cg25c*. These α chains assemble into homo- or heterotrimeric helices, forming complex extracellular networks, which underlie the structural integrity of the BM (Fessler and Fessler, 1989). Both *vkg* and *cg25c* are expressed in the hemocytes, as previously described (Le Parco et al., 1986; Yasothornsrikul et al., 1997), but neither is expressed in the tubules (Figures 3A and 3B).

Laminin is a heterotrimer of three distinct chains (α, β, and γ). In *Drosophila* there are two α chains (*wingblister* [*wb*] and *laminin A*) and single β (*laminin B1*) and γ (*laminin B2*) chains (Chi and Hui, 1988; Fessler et al., 1987; Garrison et al., 1991; Martin et al., 1999; Montell and Goodman, 1988). Martin et al. (1999) indicate that *wb* is not expressed in hemocytes or in developing tubules. However, our in situs against the other α chain, laminin A, as well as laminin B1 and B2 reveal that all three are expressed in both hemocytes and developing tubules until stage 15 when tubule expression fades (Figures S2A–S2C). Thus, both tissues express all three laminin chain types during the major phase of tubule elongation and pathfinding.

The heparin sulfate proteoglycan perlecan is encoded by *Drosophila trol.* Trol expression in hemocytes has not been reported until larval stages (Lin, 2004; Lindner et al., 2007). Accordingly our in situs showed only low levels of *trol* expression in tubule-associated hemocytes during stage 15. The tubules remain unstained throughout embryogenesis (Figure S2F). A nidogen/entactin ortholog, encoded by *CG12908* and a component of the *Drosophila* oocyte BM (Fakhouri et al., 2006), is not expressed in hemocytes or the tubules during embryogenesis (Figure S2G).

The expression patterns (Table S1) suggest laminins and collagen IV as candidate BM components that are laid down around the tubules by hemocytes. We therefore stained *pvr* mutant embryos for laminin and collagen IV. An antibody raised against the heterotrimeric laminin complex stains the BM ensheathing the tubules in both wild-type and in *pvr* mutant embryos, in which hemocyte migration is stalled and the anterior tubules extend into the posterior (Figures S2D and S2E). It therefore seems likely that, although the hemocytes might contribute to laminins surrounding the tubules, the tubules themselves deposit laminin into the BM. In contrast, the sheath of collagen IV around the developing tubules is completely lost when hemocyte migration is stalled in *pvr* mutant embryos. This is particularly clear in embryos carrying a Vkg-GFP protein trap (Morin et al., 2001); the extracellular sheath of collagen IV seen in wild-type (Figure 3C) is completely lost in *pvr* mutant embryos (Figure 3D).

We next asked whether the deposition of collagen IV is important for the normal forward extension of the anterior tubules. We approached this in two ways, first by removing collagen IV genetically and second by preventing the secretion of collagen IV either by compromising the activity of the enzyme lysyl hydroxylase (LH) (Myllyla et al., 2007) or by removing the collagen-binding ECM glycoprotein dSparc (Martinek et al., 2008). Reduction of the maternal contribution of the collagen IVs causes early and pleiotropic phenotypes in dorsoventral patterning, precluding analysis of Malpighian tubule phenotypes (Wang et al., 2008). However, embryos zygotically homozygous for a lethal P-element insertion in *vkg* (*vkg^K00236^*) exhibit a low penetrance tubule phenotype (15%, n = 233), which includes misrouting of anterior tubules to the posterior (Figure 3E). The low penetrance is most likely due to the persistence of maternal product and redundancy with the second collagen IV. However, we confirmed that misrouting is due to the reduction specifically in hemocyte-secreted collagen IV by driving *vkgRNAi* in these cells; anterior tubules in these embryos also project posteriorly (Figure 3F).

In many organisms, the deposition of mature collagen depends on lysyl hydroxylases, which catalyze the formation of hydroxylysyl residues in the Y-position of Gly-X-Y motifs of collagen α chains in the ER (Ruotsalainen et al., 2006). Genomic analysis established the existence of a lysyl hydroxylase family member in *Drosophila* (Wang et al., 2002). We identified *CG6199* as a single *Drosophila* ortholog of vertebrate *lysyl hydroxylase 3* (*plod3*), which we have named *dPlod*. dPlod is expressed in hemocytes and the sheath of collagen IV around the tubules is not secreted in embryos carrying a deficiency uncovering *dPlod* (S.B., B. Denholm, and H.S., unpublished data). Strikingly the anterior tubules misroute toward the posterior of embryos lacking dPlod (Figure 3G*).* dSparc is also expressed in hemocytes (Martinek et al., 2002), where it associates with collagen IV and facilitates its secretion. In embryos carrying a mutation in *dsparc*, collagen IV is not secreted into the BM (Martinek et al., 2008). Examination of *dsparc* mutant embryos carrying the reporter *vkg-GFP* reveals that collagen IV is expressed by hemocytes but secretion into the BM around the tubules completely fails (Figures S2H and S2I) and the anterior tubules project to the posterior (Figure 3H). Together these data show that the normal deposition by hemocytes of a collagen IV sheath around the developing anterior tubules is essential for their forward extension through the body cavity.

**Figure 4:**
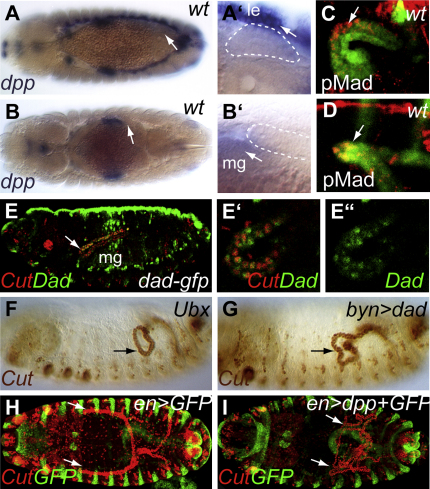
Dpp Signaling Acts as a Guidance Cue for Anterior Tubule Morphogenesis (A and B) The anterior tubules (dotted white lines in A′ and B′) develop in close proximity to *dpp*-expressing tissues (in situ for *dpp* in blue); at stage 13 adjacent to the *dpp*-expressing leading edge (le) cells (A and A′, arrow) and at stage 14 close to *dpp*-expressing midgut (mg) cells (B and B′, arrow. See also Figures S3A and S3B. (C–E) Dpp signaling is activated in a subset of anterior tubule cells. (C and D) Stage 13 (C) and 14 (D) embryos stained for phosphorylated Mad (pMad, red, Cut, green) reveal Dpp pathway activation in the kink cells of the anterior tubules (arrows). (E) The Dpp target, Dad (DadGFP, green), is activated in these anterior tubule cells (Cut, red) by stage 16 (E, arrow; magnified view in E′ and E″). (F and G) Loss of midgut Dpp signaling in *Ubx* embryos (F) or repression of target activation by driving the expression of Dad in the tubules (*bynGal4,UAS-dad*) (G) results in failure of forward projection of anterior tubules (arrows; stage 15 embryos stained for Cut, brown). (H and I) Ectopic expression of *dpp* in *engrailed*-expressing cells (green; *enGal4, UASdpp,UASgfp*) disrupts anterior tubule positioning (arrows). All four tubules bundle near their site of eversion from the hindgut (I), failing to extend anteriorly or posteriorly as in wild-type (H). See also Figure S3C. No filopodia are observed extending from kink cells (see Figures S3D–S3G and Movie S3). Anterior is to the left and dorsal is to the top in lateral perspectives.

The ECM provides mechanical support to cells and tissues but also exerts a regulatory role, controlling signaling between cells (for review, see Streuli, 1999). Collagen IV has recently been shown to modulate BMP pathway activity in *Drosophila* (Wang et al., 2008). One of the TGF-β superfamily ligands in *Drosophila*, *decapentaplegic* (*dpp*), is expressed in tissues that the anterior tubules move past as they grow out, such as the dorsal-most epidermal cells (the leading edge cells; for review, see Harden, 2002), in a band in the central midgut visceral mesoderm (Bienz, 1997) and in the gastric cecal visceral mesoderm (Panganiban et al., 1990) (Figures 4A, 4B, S3A, and S3B). We assessed whether signaling from these sources of Dpp leads to BMP pathway activation in the tubules using an antibody to phosphorylated Mad and a GFP reporter line for the pathway target Dad (Ninov et al., 2010; Weiss et al., 2010). Both reveal activation of the pathway specifically in the leading kink cells of anterior tubules, as the tubules contact the dorsal epidermis during stage 13 (Figure 4C) and the central midgut during stage 14 (Figures 4D and 4E).

In order to assess the effects of pathway activation, we made high-resolution movies of tubule cells in the region of the kink (Movie S3). We found that while none of the tubule cells develop filopodia, they all produce basal ruffles, which are active as the kink moves past the midgut (Figures S3D–S3G). Thus, it is unlikely that tubule guidance depends on the tractive force of kink cell filopodia but could result from the polarized activity of membrane lamellipodia.

We were able to show that the midgut expression of Dpp is required for normal anterior tubule pathfinding using an amorphic allele of *Ubx,* whose product lacks the homeodomain (Weinzierl et al., 1987). In such mutants, the midgut visceral mesoderm fails to express *dpp* (Reuter et al., 1990). The anterior tubules do not extend past the midgut but remain in the posterior part of the embryo (Figure 4F). This misrouting could be a secondary consequence of failure of the central midgut constriction in *Ubx* mutants (Tremml and Bienz, 1989). However, we confirmed a direct requirement for BMP pathway activation in the tubules by driving the expression of the pathway antagonist Dad (Tsuneizumi et al., 1997) specifically in this tissue. In these embryos, the anterior tubules stall at the level of the midgut, which develops normally, and remain in the posterior half of the embryo (Figure 4G). These results suggest that Dpp secreted from a band of midgut visceral mesoderm acts locally as an attractive cue for the leading (kink) cells of the anterior tubules.

We tested this hypothesis by engineering ectopic expression of Dpp in two different tissues; *engrailed*Gal4 to drive in the posterior compartment of every epidermal segment, and *caudal*Gal4 to drive in the posterior gut. In both cases, the anterior and posterior tubules extended toward the ectopic sources of *dpp* expression (Figures 4H, 4I, and S3C). Together these data show that Dpp is not only necessary but also appears to be sufficient to guide the anterior tubules as they extend.

**Figure 5:**
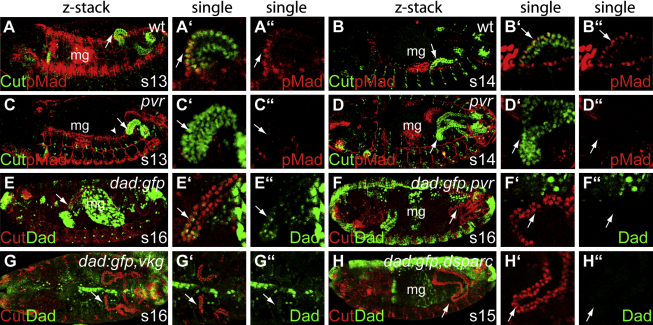
Hemocyte-Mediated Collagen IV Deposition Sensitizes Anterior Tubule Cells to Dpp Guidance Cues (A–D) Phosphorylated Mad marks out cells activated by Dpp signaling (pMad, red). (A and B) During stage 13 (A) and 14 (B) anterior tubule kink cells (Cut, green) are pMad positive (arrows). (C and D) In *pvr* mutant embryos hemocyte migration is stalled and pMad activation fails in the anterior tubules (arrows), despite their proximity to the epidermal leading edge (arrowhead) or midgut sources of Dpp (C, stage 13; D, stage 14 embryos). Note the posterior projection of tubules in (D). (E–H) In control embryos, DadGFP (green) is expressed in a subset of anterior tubule cells (Cut, red) by stage 16 (E arrows) but is absent in *pvr* mutant embryos (F, arrow). A similar phenotype is found in *vkg* (G, arrow) and *dsparc* mutant embryos (H, arrow), in which collagen IV deposition fails (Figure S2). Note tubule misrouting in mutant embryos (F–H). mg, midgut. Anterior is to the left and dorsal is to the top in lateral perspectives.

Wang et al. (2008) have shown that the interaction between Dpp and collagen IV can modify BMP signaling and suggest that collagen can act either to facilitate ligand presentation or to sequester it. In embryos lacking PDGF/VEGF signaling, the tubules remain denuded of collagen IV. We analyzed activation of the BMP pathway in *pvr* mutant embryos and found that Mad was not phosphorylated in the kink region of the anterior tubules either during stage 13 (Figures 5A and 5C) or stage 14 (Figures 5B and 5D) and that Dad-GFP was also absent from these leading cells in the mutant embryos (cf. Figures 5E and 5F).

To confirm the importance of collagen IV in the BM for BMP activation in tubule cells, we analyzed the activation of the pathway in embryos lacking the zygotic contribution of Vkg and in *dsparc* mutant embryos, in which collagen IV secretion into the BM fails (Figure S2I). As in *pvr* mutants, Dad-GFP is not expressed in the kink region of these embryos (Figures 5G and 5H). Thus, the requirement for the normal deposition of collagen IV for tubule morphogenesis is to facilitate the reception of guidance cues; tubule cells are sensitized to BMP signaling through the activity of hemocyte-secreted collagen IV.

## Discussion

As the renal tubules extend through the body cavity, two processes occur; they elongate through cell rearrangements and they make precise, guided movements with respect to other tissues. A major source of the motive force required for tubule extension is the convergent-extension movements of the tubule cells themselves (Denholm et al., 2005). As the tubules are continuous with the hindgut and thus have a fixed point proximally, these movements result in a distal-directed extensive force. We now show that in addition the normal morphogenesis of the anterior tubules depends on tissue guidance involving the coordinated activity of the PDGF/VEGF and BMP signaling pathways. Abrogation of either pathway has no effect on convergent-extension movements in the tubules but leads to failure of their normal pathfinding through the body cavity.

**Figure 6:**
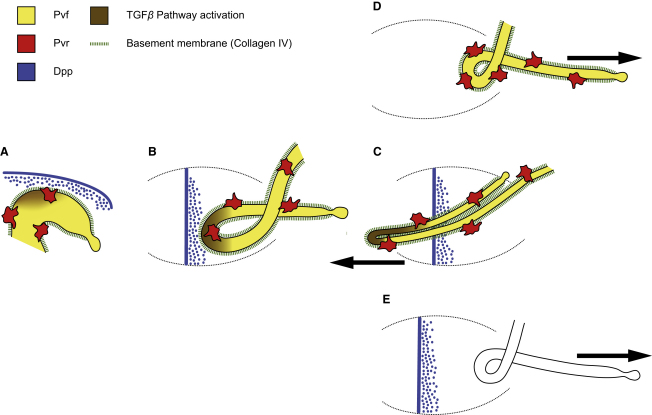
Tissue Interactions Underlie Anterior Tubule Morphogenesis (A–C) In the wild-type (stages 13 [A], 14 [B], 16 [C]) migrating hemocytes express Pvr (red) and respond to Pvf ligands (yellow) from the tubules. Hemocytes associate with the tubules, secreting BM components including collagen IV (green dashed lines). As the anterior tubules elongate (B), the most anterior region (the “kink” domain) comes to lie close to sources of Dpp (blue), first leading edge epidermal cells (A) and later a ring of midgut visceral mesoderm cells (B and C). Dpp pathway activation in the kink cells (brown) is manifest by the phosphorylation of Mad (pMad) and expression of Dad and leads to directional morphogenesis of the tubules (arrow in C), ensuring that they project forward. (D and E) In embryos in which Dpp signaling is compromised (D), hemocyte migration is stalled or collagen IV deposition is inhibited (E) Dpp pathway activation fails and the anterior tubules misdirect to the posterior (arrows). Anterior is to the left and dorsal is to the top in lateral perspectives.

PVF ligands expressed by the tubules attract migrating hemocytes to form short-term associations with them, during which hemocytes secrete components of the BM. The presence of collagen IV in the matrix ensheathing anterior tubule cells primes their response to local sources of the BMP pathway ligand, Dpp. Thus, interference with hemocyte secretion of collagen IV, whether by preventing hemocyte migration, by preventing their attraction to the tubules, or by abrogating hemocyte expression and/or processing of collagen IV, results in failure of BMP pathway activation in tubule cells and consequent misrouting of the anterior tubules. The tissue interactions that govern the guided outgrowth of the anterior tubules are summarized in Figure 6.

As the tubules elongate, a distinct but dynamic subset of cells in the kink region responds sequentially to Dpp guidance cues from dorsal epidermal cells, the midgut, and, more anteriorly, gastric cecal visceral mesoderm and leads forward extension. Activation of the pathway targets, pMad and Dad, in these leading cells ensures that as the tubules project through the body cavity they take a stereotypical route. Loss of Dpp expression in the midgut or repression of BMP signaling in the tubules leads to stalling of their forward movement. Misexpression of Dpp is sufficient to cause tubule misrouting, in which the kink regions project toward the ectopic source. In accordance with our findings, Jack and Myette (1999) describe defective tubule morphogenesis in embryos lacking the BMP receptors *thick veins* (type 1) or *punt* (type II), as well as in embryos mutant for *schnurri*, which encodes a pathway transcriptional regulator shown to be active during embryogenesis (Arora et al., 1995).

Strikingly only cells in the kink show pathway activation. Our evidence suggests that leading kink cells respond directionally to local gradients of Dpp and that they receive the highest level of ligand, which would account for the restricted domain of activation. However, as the kink region extends beyond the Dpp source, more posterior cells experience high levels of signal but show no pathway activation, indicating that other factors must differentiate between the leading and trailing cells. Segregation into leading and following populations is a common feature of collective cell migration and tubule branching and extension during organogenesis (for review, see Andrew and Ewald, 2010; Rorth, 2009). Leading cells in outgrowing *Drosophila* trachea, migrating border cells and mammalian ureteric bud formation show distinct patterns of gene expression, respond differentially to external signals, and may repress pathway activation in their neighbors (Affolter et al., 1994; Bianco et al., 2007; Chi et al., 2009; Ghabrial and Krasnow, 2006; Shakya et al., 2005; Vincent et al., 1997). Thus, tubule kink cells could themselves restrict the domain of pathway response.

As well as their roles in determining cell fate, survival, and growth in *Drosophila* (for review, see Affolter and Basler, 2007), TGF-β superfamily signals regulate tissue morphogenesis and have been shown to influence the invasive behavior of metastatic tumors (for review, see Leivonen and Kahari, 2007; Wu and Hill, 2009). We show here, through loss- and gain-of-function analysis, that Dpp also acts as a chemoattractant during organogenesis to determine the path of renal tubule extension though the body cavity. TGF-β superfamily signaling can induce epithelial-to-mesenchymal (EMT) transition through the expression of Snail- and ZEB-family members, which act to repress cell adhesion and polarity (Bryant and Mostov, 2008; Yang and Weinberg, 2008), leading to increased motility and, in the case of cancers, to single-cell metastatic activity (Giampieri et al., 2009). Such changes in kink cells could explain their role in pathfinding. However, recent evidence suggests that collective cell migration of epithelial tissues can occur without full EMT (Revenu and Gilmour, 2009; Wicki et al., 2006; Vasilyev et al., 2009) and kink cells remain polarized, ensheathed in ECM during tubule elongation.

Ninov et al. (2010) have shown that pathway activation through pMAD leads to increased actin dynamics and E-cadherin turnover in outgrowing histoblasts, resulting in reduced cell adhesion and enhanced cell motility through filopodial/lamellipodial extensions. Our results reveal similar lamellipodial extensions in kink cells, in line with Vasilyev et al. (2009), who demonstrated directional basal lamellipodia in cells of the extending pronephric tubules of zebrafish. It is possible that the production of lamellipodia and tubule navigation also depends on Mad-independent effects on cytoskeletal regulators such as cdc42 (Ricos et al., 1999) and Rho1, Rac, and LIM kinase (Ng, 2008).

Our analysis reveals that deposition of ECM is a prerequisite for BMP signaling in tubule guidance. TGF-β/BMP signaling can be modified both by soluble ECM components such as HSPGs (Hacker et al., 2005) and also by architectural, fibrillar elements (Ramirez and Rifkin, 2009). Our evidence indicates that for normal tubule outgrowth collagen IV is the crucial component of the BM; it is deposited before tubule elongation (cf. perlecan deposited after elongation), is uniquely contributed by the hemocytes (the tubules express laminins as well as the hemocytes), and the effects of collagen IV loss of function mimic the failure of hemocyte migration to the tubules (whether in *collagen IV* mutants or in embryos lacking the function of lysyl hydroxylase or dSparc, factors that are required for normal collagen IV processing and deposition) (Martinek et al., 2008; Myllyla et al., 2007).

Collagen IV sharpens the dorsoventral gradient of BMP signaling in early *Drosophila* embryos through enhanced ligand-mediated activation (Wang et al., 2008), which depends on a conserved BMP-binding domain in the C-terminal region of collagen IV. Wang et al. (2008) propose a two-step process in which the binding of Dpp/Screw ligand hetereodimers to collagen IV facilitates the formation of a complex between Dpp/Scw dimers, Sog, and Tsg. Tolloid cleavage of the complex releases ligand dimers, which become active on rebinding to collagen IV dorsally where Sog is absent. We now show that basement membrane collagen IV also acts during organogenesis to facilitate BMP signaling in a specialized region of tubule cells. Whereas the mechanism of activation could be as outlined by Wang et al. (2008), early requirements for Dpp signaling in tubule development (Hatton-Ellis et al., 2007) complicate further analysis.

Although the forward extension of the anterior tubules is important for their morphogenesis, it is likely that other factors regulate their navigation through the body cavity. The kink region dips ventrally and the distal tips extend dorsally late in embryogenesis so that specialized cells at the distal tip (Hoch et al., 1994; Skaer, 1989) contact dorsal structures. Further, morphogenesis of the posterior tubules is unaffected by the repression of BMP signaling; they migrate posteriorly, crossing the hindgut and adopt their normal position in the body cavity, with their tip cells contacting hindgut visceral nerves (Hoch et al., 1994). It is probable that the coordination of multiple inputs controls the morphogenetic movements of all four tubules.

We have highlighted the importance of multiple tissue interactions in the outgrowth of *Drosophila* renal tubules, between the tubules and hemocytes, and, as a consequence of this interaction, with guidepost tissues such as the midgut visceral mesoderm. Similar interactions occur during the specification and recruitment of renal tubule cells, in the branching of the ureteric bud and in the formation of the glomerulus (Vize et al., 2003). In vertebrate nephrogenesis kidney medullary and cortical tubules extend, taking up stereotypical positions with respect to blood vessels, with which they later interact to maintain tissue homeostasis. TGF-β superfamily signaling plays multiple roles early in vertebrate kidney development (Luo et al., 1995; for review, see Cheng and Grande, 2002; Vize et al., 2003) so that analysis of signaling during renal tubule morphogenesis requires conditional alleles or specialized reagents. Such studies reveal requirements for TGF-β superfamily signaling in the morphogenesis of the pronephric tubules and duct in *Xenopus* (Bracken et al., 2008), and for the maintenance and morphogenesis of mammalian nephrogenic mesenchyme (Oxburgh et al., 2004). VEGF is expressed in early renal mesenchyme and ureteric bud (Loughna et al., 1997) and later in glomeruli (Breier et al., 1992), where it is essential for glomerular capillary growth (Carmeliet, 1999; Gerber et al., 1999; Kitamoto et al., 1997). It will be exciting to discover whether a combination of VEGF/PDGF ligands in renal tissues and spatially restricted TGF-β superfamily guidance cues underpins the coordinated morphogenesis of these spatially linked renal/blood systems, as we have shown occurs in *Drosophila*.

## Experimental Procedures

### *Drosophila* Genetics

Fly stocks were maintained on cornmeal agar according to standard protocols (Greenspan, 1997). Crosses were carried out at 25°C unless otherwise stated. For strains, see Supplemental Experimental Procedures.

### Immunostaining and In Situ Hybridization

Immunostaining was performed using standard techniques with the antibodies listed (see Supplemental Experimental Procedures). When required, we performed an amplification step using streptavidin-conjugated FITC or Cy3. For anti-pMad staining, we employed an additional 20 min amplification using the tyramide system (PerkinElmer).

RNA localization was performed by in situ hybridization using DIG-labeled RNA probes generated by in vitro transcription from DNA templates (see Supplemental Experimental Procedures). Hybridization and staining of embryos was according to standard protocols (Nagaso et al., 2001; Tautz and Pfeifle, 1989).

### In Vivo Imaging

Embryos were dechorionated in bleach, and early stage 13 embryos transferred onto a droplet of Voltalef 3S oil on a glass slide and mounted with a raised glass coverslip. Live imaging was performed using a Leica SP5 scanning laser microscope. To photoconvert Kaede, cells in the kink region were exposed to a 55–85 s UV pulse.

## Supplemental Information

Document S1. Three Figures, One Table, and Supplemental Experimental Procedures

Movie S1. Cells of the Kink Region Remain a Relatively Stable Population during Migration Past the Midgut Time-lapse recording of late stage 13 to 16 *CtBGal4, UAS-Stinger-RFP* embryo from a lateral perspective shows that many cells (nuclei, red) in the kink remain in this region as the tubule extends past the midgut.

Movie S2. Haemocyte Association with the Tubules Is Very Dynamic Time-lapse recording of a stage 13 to 16 *srpHemoGal4, UASmCherry; cutB-gfp* embryo from a lateral perspective showing dynamic interactions between the migrating haemocytes (red) and an elongating anterior tubule (green) as it extends past the midgut towards the head.

Movie S3. No Basal Filopodia Extend from Kink Cells during Tubule Pathfinding Time-lapse recording of a stage 13 to 16 *cutBGal4, UASmoesinGFP* tubule from a lateral perspective showing dynamic basal lamellipodia arising from kink and other tubule cells but no filopodia.
