# Supplementary material for: Hemocyte-Secreted Type IV Collagen Enhances BMP Signaling to Guide Renal Tubule Morphogenesis in Drosophila
Source: Dev Cell. 2010 Aug 17;19(2):296–306. doi: 10.1016/j.devcel.2010.07.019 (PMC2941037; doi:10.1016/j.devcel.2010.07.019)
Supplement: Document S1. Three Figures, One Table, and Supplemental Experimental Procedures [file mmc1.pdf]

Developmental Cell *19*

Supplemental Information

Hemocyte-Secreted Type IV Collagen Enhances BMP Signaling to Guide Renal Tubule Morphogenesis in *Drosophila*

Stephanie Bunt, Clare Hooley, Nan Hu, Catherine Scahill, Helen Weavers, and Helen Skaer

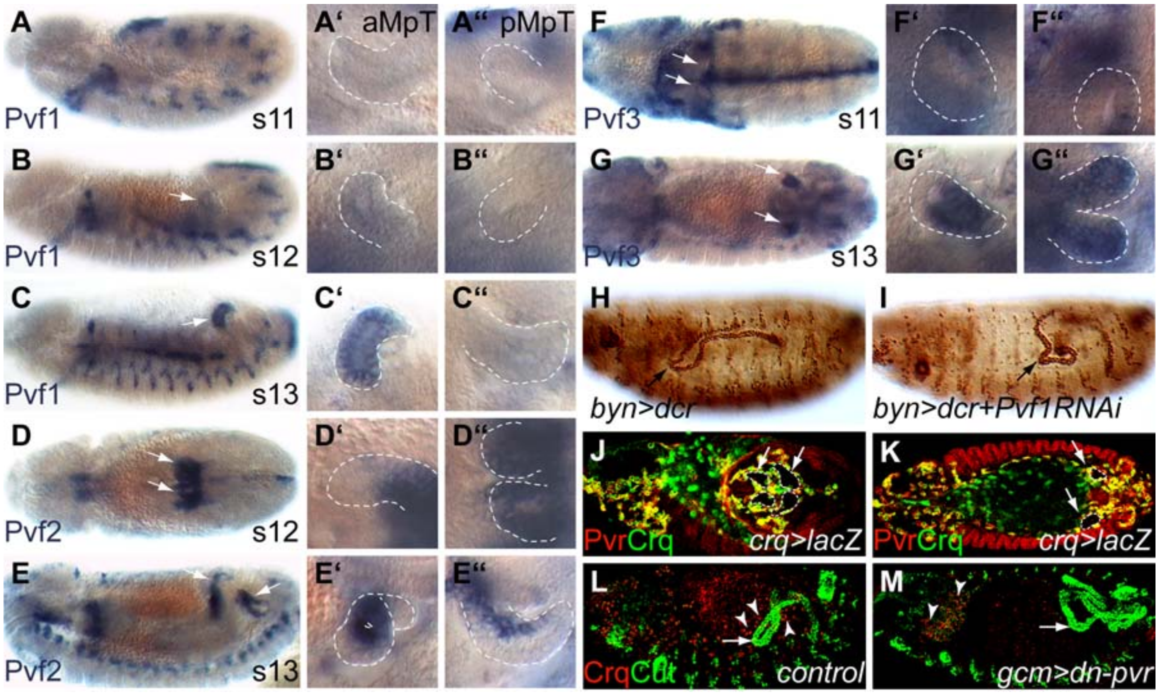

Figure S1, related to Figure 2. Pvf ligands expressed in Malpighian tubules attract a subset of migrating haemocytes, which are required for anterior tubule morphogenesis
(A-G) *in situ* hybridisation for PDGF/VEGF ligands, Pvf 1, 2 and 3, with magnified view of anterior (A-G') and posterior (A-G") tubules (outlined by dashed white lines). (A-C) *pvf1* is expressed in the kink and distal regions of anterior tubules from late germ-band extension (stage 11, A), strengthening during stage 12 (B, arrow) to express strongly from stage 13 (C, arrow). (D, E) *pvf2* expression persists through stage 12 (D, arrows) and 13 (E, arrows), in the proximal region of all tubules. (F, G) *pvf3* is expressed in the distal region of all tubules from stage 11 (F, arrows), persisting through stage 13 (G, arrows). (H,I) Knock down of Pvf1 in the tubules (*bynGal4>UAS-dicer; UAS-pvf1RNAi*) results in the anterior tubules (stained for Cut) misrouting to the posterior (I, compare with *bynGal4>UAS-dicer* control, H), reminiscent of the *pvf1* mutant phenotype (Fig 2H, penetrance 56%) (J, K) Pvr-expressing cells (red) associated with the tubules (outlined in J, K) also express the haemocyte marker Croquemort (*crqGal4, UASLacZ*; green, arrows). (L, M) Expressing a dominant-negative form of the receptor, Pvr, in haemocytes

(*gcmGal4, UASdnpvr*) stalls their migration (arrowheads in M), preventing association with the tubules. The anterior tubules fail to project forwards in these embryos (*cf.* M, with control embryo L, arrows: stage 14 embryos).

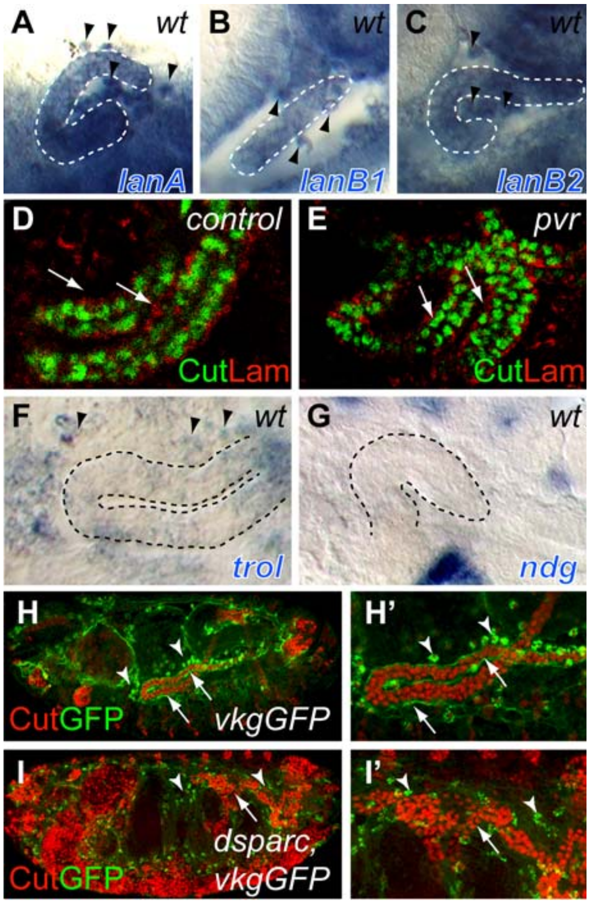

Figure S2, related to Figure 3. Haemocytes secrete many components of the extracellular matrix, and the secretion of Collagen IV can be repressed
(A-E) The three laminin chains, A, B1 and B2 are expressed in the tubules and in haemocytes that associate with them (A-C, white dotted lines and arrowheads,). (A-C) Stage 14 embryos, in situ hybridisation against *laminin A* (*A*), *B1* (B) and *B2* (C). Laminin protein (red) is detected in the tubule BM in control (D) and in *pvr* mutant (E) embryos, in which haemocyte migration is stalled (D, E, stage 15, laminin, red and arrows). (F) *in situ* hybridisation against the heparin sulphate proteoglycan *perlecan* (encoded by *trol*) shows low levels of expression in haemocytes by stage 15 (arrowheads). (G) The single *Drosophila* orthologue of *nidogen/entactin* (*CG12908*) is not expressed in tubule cells or haemocytes (stage 14 embryo). F, G dotted lines indicate a single tubule. (H, I) The secretion of Collagen IV can be repressed.
(H) Vkg GFP is expressed by haemocytes (arrowheads) and secreted into the BM ensheathing the tubules (H, H' arrows; stage 15 embryo). In *d-sparc* mutant embryos of a similar age (I) VkgGFP expressed in the haemocytes (arrowheads) is not secreted around the tubules (I, I' arrows).

Table S1.

Basement membrane gene (protein)	Expression in tubule-associated haemocytes	Expression in developing Malpighian tubules
viking (Collagen IV)	+	-
cg25c (Collagen IV)	+	-
wingblister (Laminin-α 1,2)$^{\$}$	-	-
laminin A (Laminin-α 3,5)	+	+
laminin B1	+	+
laminin B2	+	+
*trol (*Perlecan)	+*	-
CG12908 (Nidogen/Entactin)	-	-

$ from (Martin et al., 1999)

* expression starts only after tubule elongation

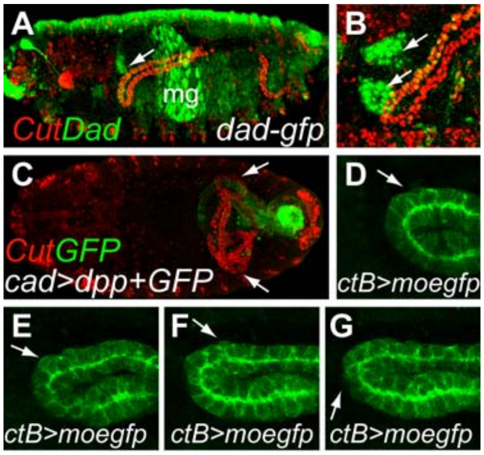

Figure S3, related to Figure 4. The anterior tubules respond to Dpp signalling from surrounding tissues
(A, B, stage 16 embryos) Dpp signalling is active in the anteriorly-located gastric caeca. The Dpp target, Dad (DadGFP green), is activated in the gastric caeca (arrows; magnified embryo in B), close to the migrating anterior MpTs (red). (C) Ectopic expression of *dpp* in *caudal*-expressing cells (shown in green; *cadGal4, UASdpp,UASgfp*) disrupts anterior tubule positioning (stained for Cut, red, arrows). All four tubules bundle near the source

of *caudal*-driven Dpp expression in the hindgut (I), failing to extend anteriorly or posteriorly. (D-G) Labelling actin in tubule cells reveals that they produce basal ruffles or lamellipodia (arrows) but no filopodial extensions as the tubules elongate (stills from Suppl movie 3 of *CtBGal4>moesinGFP* embryo).

SUPPLEMENTAL EXPERIMENTAL PROCEDURES

Drosophila Genetics

Wild-type analysis was performed on *OregonR* embryos. The following alleles were used for mutant analysis: pvr^{C02195}, $pvf1^{G0146}$, $Ubx^{9.22}$, vkg^{K00236}, *d-sparc,* and *Df(3L)vin66. FM7c-ftz-lacZ, CyO-wg-lacZ* and *TM3-ftz-lacZ* blue balancers were used to identify homozygous embryos except for $Ubx^{9.22}$ (balanced over TM1), where a homeotic transformation which results in multiple anterior spiracles was used to positively identify homozygous embryos when stained for anti-Cut. We used the GAL4 system (Brand and Perrimon, 1993) for mis-expression experiments with the following lines: *byn-Gal4 (gift of R. Reuter), crq-Gal4 (gift of B. Olofsson), cutB-Gal4 (gift of V. Sudarsan), en-Gal4 (gift of A. Brand), gcm-Gal4 (gift of U. Tepass), srpHemo-Gal4 (gift of W. Wood), UAS-dad (gift of T. Tabata), UAS-DN-pvr (gift of P. Rorth), UAS-dicer (gift of S. Thor) , UAS-pvf1RNAi, UAS-collagen IV RNAi, UAS-viking RNAi (all from VDRC). UAS-gfp, UAS-βgal,* $UAS\text{-}rac^{V12}$*, UAS-dpp, cad-Gal4,UAS-moesinGFP and UAS-StingerRFP were obtained from the Bloomington Stock Centre, Indiana.* The following reporter lines were used: *dad-gfp(nuclear) (gift of M. Affolter)* and *vkg-gfp (gift of W. Chia).* For strong hemocyte expression we crossed *crq-Gal4* into *srpHemo-Gal4*. To visualise the haemocytes and MpTs *in vivo* we used a stock homozygous for *srpHemo-Gal4* recombined with *UAS-mCherry* on the second (kindly provided by W. Wood), and the *cutB* promoter region attached to *GFP* on the third (K. Campbell & HS).

Immunohistochemistry and *in situ* hybridisation

For immunostaining the following antibodies were used: mouse anti-Cut (1:200, DSHB), rabbit anti-Pvr (1:1000, D. Montell), mouse anti-Peroxidasin (1:1500, J. Fessler), rabbit anti-GFP (1:500, Abcam,), rabbit anti-phosphorylated Mad (1:2000, P. ten Dijke), rabbit anti-βgalactosidase (1:10000, Cappel), rabbit anti-Croquemort (1:1000, D. Montell), and rabbit anti-Laminin (1:1000, J. Fessler). Biotinylated secondary antibodies were used in conjunction with the Vector Elite ABC kit (Vector Laboratories) for DAB staining or fluorescent secondary antibodies conjugated to FITC or Cy3 (1:200, Jackson ImmunoResearch). Embryos were collected from agar apple juice plates, dechorionated in bleach, fixed for 20 minutes in 4% paraformaldehyde, and de-vitellinised in a 1:1 heptane:methanol mixture by vigorous shaking. For anti-pMad staining, embryos were fixed in 8% formaldehyde. cDNA was obtained from the *Drosophila* Genome Resource Centre for the following genes: *pvf1* (clone LD28763), *pvf2* (RH40211), *pvf3* (RE18107), *vkg* (RE68619), *cg25c* (GM04010), *lanA* (LD24387), and *lanB2* (LD15803). *dpp* cDNA was kindly provided by Hilary Ashe. The following primer pairs were used (Sigma-Genosys) to generate a DNA template from genomic DNA using standard PCR conditions: *lanB1* (5'-GCG GCT TAT CGT GGT CAT TGT, 5'-GCA GCT TCG TCA TCT GGA TCC), *ndg* (5'-GCC CGT TTC CTG AGC GAA AGC, 5'-CCA ACA CAC GCT GCT CCT CGG), *trol* (5'-CGC AGT CAC CGG TAT CGC TGT, 5'-CCC ACG CCA GAC ACC GTA CCT). For light microscope analysis, embryos were either dehydrated and cleared in Histoclear (Fisher-Scientific) before mounting in DPX (BDH

Laboratory Supplies), or alternatively mounted in 80% glycerol to facilitate embryo rolling, and then imaged using a Zeiss Axioplan with a JCB KY-F55B digital camera. For fluorescent analysis, all embryos were mounted in Vectashield (Vector Laboratories) and imaged using a Leica Sp1 or SP5 scanning laser microscope and LSM software. For live-imaging, time-lapse series were assembled using ImageJ imaging software (NIH).

SUPPLEMENTAL REFERENCES

Brand, A. H., and Perrimon, N. (1993). Targeted gene expression as a means of altering cell fates and generating dominant phenotypes. Development *118*, 401-415.

Martin, D., Zusman, S., Li, X., Williams, E. L., Khare, N., DaRocha, S., Chiquet-Ehrismann, R., and Baumgartner, S. (1999). wing blister, a new Drosophila laminin alpha chain required for cell adhesion and migration during embryonic and imaginal development. J Cell Biol *145*, 191-201.
