# Supplementary figures and images for: Hemocyte-Secreted Type IV Collagen Enhances BMP Signaling to Guide Renal Tubule Morphogenesis in Drosophila
Source: Dev Cell. 2010 Aug 17;19(2):296–306. doi: 10.1016/j.devcel.2010.07.019 (PMC2941037; doi:10.1016/j.devcel.2010.07.019)

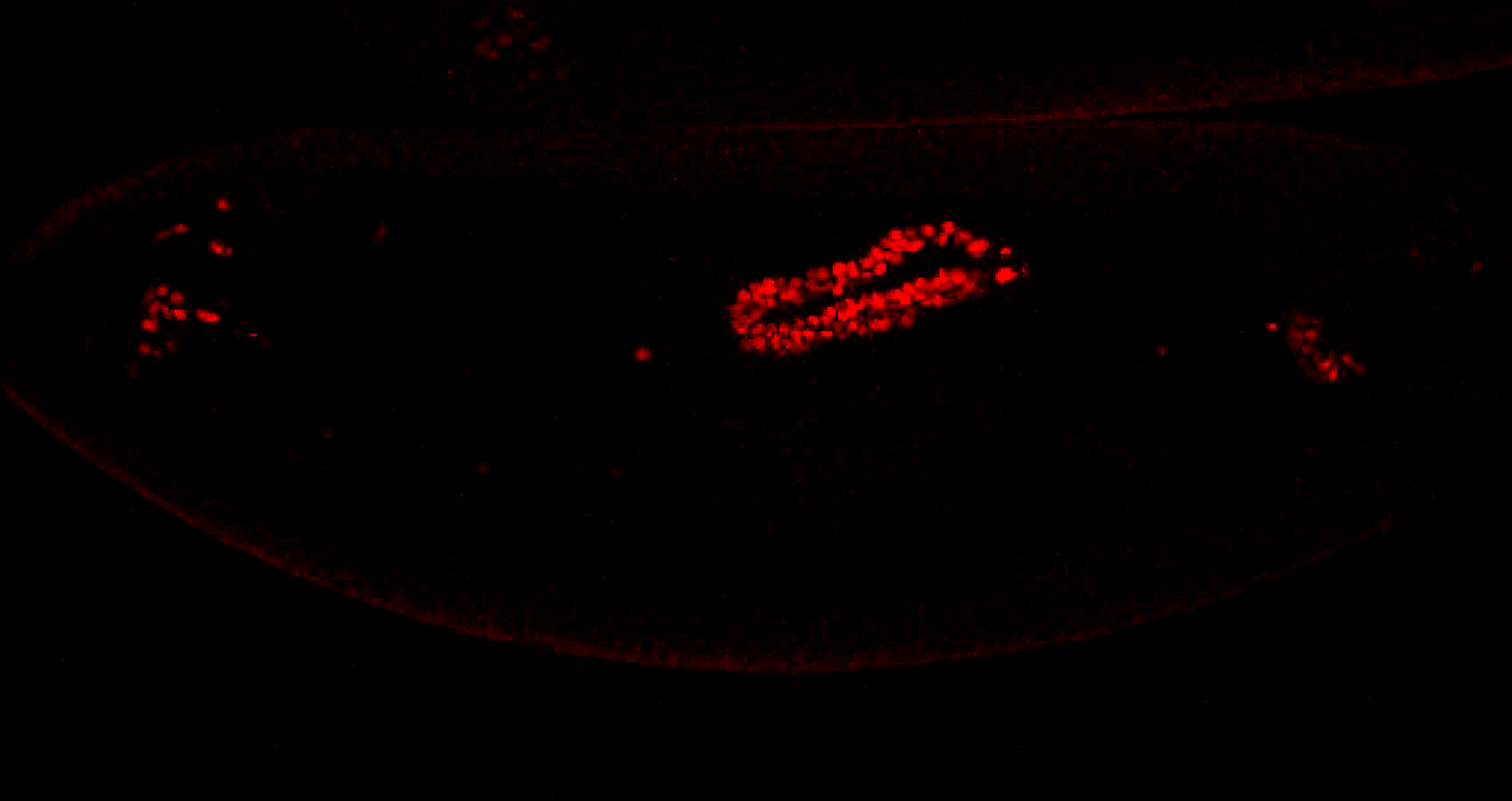

Supplement: Movie S1. Cells of the Kink Region Remain a Relatively Stable Population during Migration Past the Midgut — Time-lapse recording of late stage 13 to 16 CtBGal4, UAS-Stinger-RFP embryo from a lateral perspective shows that many cells (nuclei, red) in the kink remain in this region as the tubule extends past the midgut. [file mmc2.jpg]

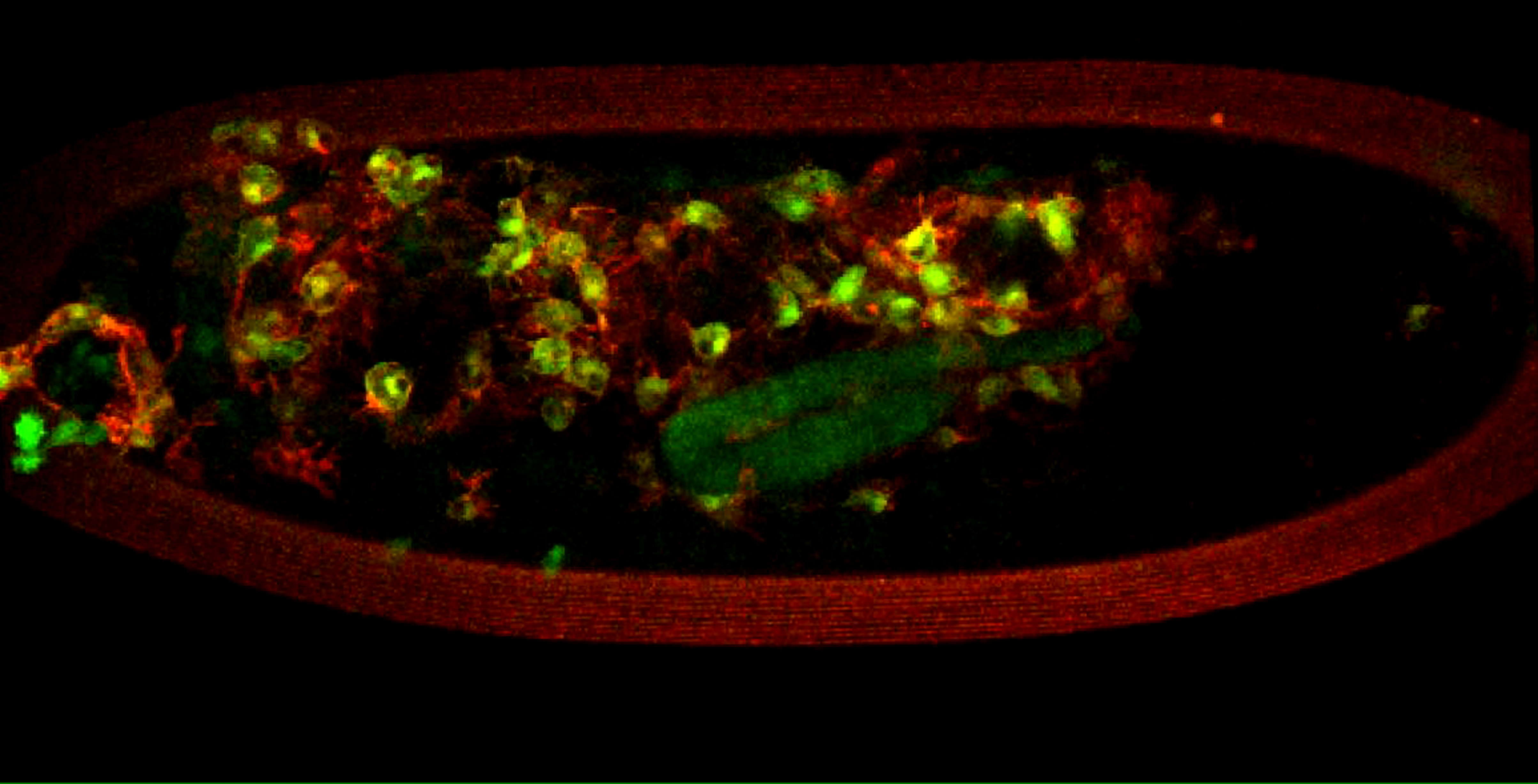

Supplement: Movie S2. Haemocyte Association with the Tubules Is Very Dynamic — Time-lapse recording of a stage 13 to 16 srpHemoGal4, UASmCherry; cutB-gfp embryo from a lateral perspective showing dynamic interactions between the migrating haemocytes (red) and an elongating anterior tubule (green) as it extends past the midgut towards the head. [file mmc3.jpg]
